# Supplementary material for: Nuclear chromosome locations dictate segregation error frequencies
Source: Nature. 2022 Jul 13;607(7919):604–9. doi: 10.1038/s41586-022-04938-0 (PMC9300461; doi:10.1038/s41586-022-04938-0)
Supplement: Supplementary file 1 — Reporting Summary [file 41586_2022_4938_MOESM1_ESM.pdf]

nature portfolio

Corresponding author(s): Geert J.P.L. Kops

Last updated by author(s): Jun 17, 2022

Reporting Summary

Nature Portfolio wishes to improve the reproducibility of the work that we publish. This form provides structure for consistency and transparency in reporting. For further information on Nature Portfolio policies, see our Editorial Policies and the Editorial Policy Checklist.

Statistics

For all statistical analyses, confirm that the following items are present in the figure legend, table legend, main text, or Methods section.

n/a	Confirmed	
☐	☒	The exact sample size (*n*) for each experimental group/condition, given as a discrete number and unit of measurement
☐	☒	A statement on whether measurements were taken from distinct samples or whether the same sample was measured repeatedly
☐	☒	The statistical test(s) used AND whether they are one- or two-sided<br>*Only common tests should be described solely by name; describe more complex techniques in the Methods section.*
☒	☐	A description of all covariates tested
☐	☒	A description of any assumptions or corrections, such as tests of normality and adjustment for multiple comparisons
☐	☒	A full description of the statistical parameters including central tendency (e.g. means) or other basic estimates (e.g. regression coefficient) AND variation (e.g. standard deviation) or associated estimates of uncertainty (e.g. confidence intervals)
☐	☒	For null hypothesis testing, the test statistic (e.g. *F*, *t*, *r*) with confidence intervals, effect sizes, degrees of freedom and *P* value noted<br>*Give P values as exact values whenever suitable.*
☒	☐	For Bayesian analysis, information on the choice of priors and Markov chain Monte Carlo settings
☒	☐	For hierarchical and complex designs, identification of the appropriate level for tests and full reporting of outcomes
☐	☒	Estimates of effect sizes (e.g. Cohen's *d*, Pearson's *r*), indicating how they were calculated

Our web collection on statistics for biologists contains articles on many of the points above.

Software and code

Policy information about availability of computer code

Data collection	Imaging data were collected using SoftWorx (Applied Precision/GE Healthcare, version 6.5.2), NIS-Elements (Nikon, version 5.30.04), Prairie View (version 5.4.64.500); Imspector (Abberior Instruments GmbH, version 16.3) or ZEN (Zeiss, version 3.3)<br>Flow cytometric data were collected using FACSDiva (BD Biosciences, version 9.0.1)<br>Single-end sequencing was performed on a Nextseq500 (50bp or 75bp) or NextSeq2000 (100bp) (Illumina, San Diego, CA, USA)
Data analysis	Raw reads were mapped to hg38 using bwa aln (version 0.7.12). Only reads starting with an NLAIII cutter site and with unique chromosome coordinates plus unique UMI were kept using python (version 2.7.5).<br>cutadapt (version 1.16)<br>bowtie2 (version 2.3.4)<br>Code to generate figures from raw sequencing data can be found at https://github.com/sjklaasen/scKaryo-seq.git.<br><br>Sequencing data were visualized and copy numbers were determined using Aneufinder (version 1.2.0) in RStudio (version 1.4.1717).<br><br>Graphpad Prism 8 was used for statistical analyses and data visualization (version 8.4.3)<br><br>Fiji ImageJ was used for imaging analyses (version 2.0.0)<br><br>FACS data was analyzed on Flowjo (version 10.6.1)<br><br>For kinetochore tracking experiments:<br>Data analysis and tracking: Fiji ImageJ (version 1.53f51/1.53s30/1.53r; Low light)<br>tracking tool plugin (version 0.10); Bio Format plugin (version 6.7.0); ImarisViewer (version 9.8.0)

Quantification: MatlabR2021a (version 9.10.0)

For manuscripts utilizing custom algorithms or software that are central to the research but not yet described in published literature, software must be made available to editors and reviewers. We strongly encourage code deposition in a community repository (e.g. GitHub). See the Nature Portfolio guidelines for submitting code & software for further information.

Data

Policy information about availability of data

All manuscripts must include a data availability statement. This statement should provide the following information, where applicable:

- Accession codes, unique identifiers, or web links for publicly available datasets
- A description of any restrictions on data availability
- For clinical datasets or third party data, please ensure that the statement adheres to our policy

Raw sequencing data can be found at the European Nucleotide Archive: PRJEB52892.

Source data are available at https://doi.org/10.6084/m9.figshare.19779736.

Previously published Dam- and Mad-Lamin B1 sequencing data for RPE1-hTERT can be found at GSM3904483, GSM3904484, GSM3904548 and GSM3904549, for hESC at GSM557443 and GSM557444, for TIG3-hTERT at GSM2030834, for HeLa at E-MTAB-6888, for K562 at GSM1612855, GSM1612856 and for HT1080 at GSM984848.

Field-specific reporting

Please select the one below that is the best fit for your research. If you are not sure, read the appropriate sections before making your selection.

☒ Life sciences ☐ Behavioural & social sciences ☐ Ecological, evolutionary & environmental sciences

For a reference copy of the document with all sections, see nature.com/documents/nr-reporting-summary-flat.pdf

Life sciences study design

All studies must disclose on these points even when the disclosure is negative.

Sample size	No statistical methods were used to predetermine sample size. Between 20-200 cells were analyzed per replicate, depending on type of experiment (live cell tracking, FISH, sequencing). The size of the sample was chosen to offer sufficient statistical power.
Data exclusions	scKaryo-seq data was excluded manually if copy number states could not be easily discerned or did not fit ploidy state after FACS. Micronuclei were excluded from single MN-seq analysis if they contained five or more chromosomes.
Replication	All attempts of replication were successful. Experiments were repeated at least twice.
Randomization	No randomization was performed. To counter batch effects, both conditions were usually sorted into the same plate.
Blinding	Data collection during FISH imaging data collection was blinded by only looking at the DAPI signal when selecting cells. For imaging and sequencing experiments, blinding was not possible because conditions could be easily identified from the sequencing or imaging data itself.

Reporting for specific materials, systems and methods

We require information from authors about some types of materials, experimental systems and methods used in many studies. Here, indicate whether each material, system or method listed is relevant to your study. If you are not sure if a list item applies to your research, read the appropriate section before selecting a response.

Materials & experimental systems

n/a	Involved in the study	
☒	☐	Antibodies
☐	☒	Eukaryotic cell lines
☒	☐	Palaeontology and archaeology
☒	☐	Animals and other organisms
☒	☐	Human research participants
☒	☐	Clinical data
☒	☐	Dual use research of concern

Methods

n/a	Involved in the study	
☒	☐	ChIP-seq
☐	☒	Flow cytometry
☒	☐	MRI-based neuroimaging

Eukaryotic cell lines

Policy information about cell lines

Cell line source(s)	RPE1-hTERT cells were a gift from the Prasad Jallepalli lab.<br>HeLa cells were a gift from the Michiel Vermeulen lab.<br>BJ-hTERT cells were a gift from the Rene Medema lab.<br>U2OS cells were a gift from the Susanne Lens lab.<br>DLD1 cells were a gift from the Daniella Cimini lab.<br>Human intestinal organoids, HCT116, Caco-2, HT-29 and Widr cell lines were a gift from the Hans Clevers lab.<br>RPE1-hTERT CENPA-GFP and Centrin1-GFP cells were a gift from the Alexey Khodjakov lab.<br>U2OS CENPA-GFP mCherry-α-tubulin photoactivatable-GFP-α-tubulin were a gift from the Helder Maiato lab.<br>HT1080 cells containing a LacO-array in chromosome 11 were a gift from the Wendy Bickmore lab.
Authentication	RPE1, BJ and HCT116 cell lines were verified based on the karyotypes as determined by scKaryo-seq.
Mycoplasma contamination	All lines were tested negative for mycoplasma.
Commonly misidentified lines<br>(See ICLAC register)	No commonly misidentified lines were used.

Flow Cytometry

Plots

Confirm that:

☒ The axis labels state the marker and fluorochrome used (e.g. CD4-FITC).

☒ The axis scales are clearly visible. Include numbers along axes only for bottom left plot of group (a 'group' is an analysis of identical markers).

☒ All plots are contour plots with outliers or pseudocolor plots.

☒ A numerical value for number of cells or percentage (with statistics) is provided.

Methodology

Sample preparation	Single G1 nuclei of RPE1-hTERT Flp-in cells or single nuclei of BJ-hTERT cells were isolated as described before (Bolhaqueiro et al., 2019 (Nat. Gen.). In short, cells were treated for around 15 min on ice with a nuclear staining buffer containing 100 mM Tris-HCl pH 7.5, 154 mM NaCl, 1 mM CaCl2, 0.5 mM MgCl2, 0.2% BSA, 0.1% NP40 (v/v), 1 ug/mL Hoechst 34580.<br><br>Small intestinal cells treated with EdU were fixed using 70% ice-cold ethanol. Ethanol was removed by one wash with PBS and cells were incubated for 10 min with the Click-iT reaction cocktail (see Click-iT EdU proliferation assay). The reaction cocktail was washed away and replaced with a PBS/DAPI mix. Single G1 nuclei in case of ZM447439 or EdU-positive G1 cells were sorted in 384-well plates.<br><br>For MN-seq, cells were incubated on ice for 30 min under light with PBS/2% FBS and 12.5 µg/ml EMA (Thermofisher). EMA was washed 4x using PBS and (micro)nuclei were harvested from the cells using the same nuclear staining buffer used for scKaryo-seq. EMA-negative and Hoechst-positive (micro)nuclei were sorted in bulk in a PCR strip containing mineral oil and stored at -20 °C for further processing.
Instrument	BD FACSJazz (cat. num. 655489), FACSAria II SORP (serial number P58000001) and FACSAria FUSION SORP (serial number R658282P4001) (BD, Franklin Lakes, NJ, USA)
Software	Flow cytometric data were collected using FACSDiva (BD Biosciences, version 9.0.1)<br>Flowjo (version 10.6.1)
Cell population abundance	Single cell or nucleus sorting purity was not explicitly determined, because a higher purity would only increase the percentage of aneuploid cells, but not influence results. Micronucleus sorting purity was visually confirmed after sorting using microscopy.
Gating strategy	FSC/SSC and Hoechst were used to gate for cells and singlets (Extended Data Fig. 1a). EdU-positive G1 cells were selected based on EdU and DAPI-staining (Extended Data Fig. 2g). Micronuclei with a Hoechst intensity 10x below G1 were gated (Fig. 2a and Extended Data Fig. 4c).

☒ Tick this box to confirm that a figure exemplifying the gating strategy is provided in the Supplementary Information.
